# Utilisation des méthodes contraceptives modernes en République Démocratique du Congo: prévalence et barrières dans la zone de santé de Dibindi à Mbuji-Mayi

**DOI:** 10.11604/pamj.2017.26.199.10897

**Published:** 2017-04-13

**Authors:** Abel Mukengeshayi Ntambue, Rachel Ngalula Tshiala, Françoise Kaj Malonga, Tabitha Mpoyi Ilunga, Josaphat Mulumba Kamonayi, Simon Tshimankinda Kazadi, Charles Matungu Matungulu, Angel Nkola Musau, Diese Mulamba, Michèle Dramaix-Wilmet, Philippe Donnen

**Affiliations:** 1 Unité d’Epidémiologie et de Santé de la Mère, du Nouveau-né et de l’Enfant, Ecole de Santé Publique, Université de Lubumbashi, Lubumbashi, RDC; 2 Institut Supérieur des Techniques Médicales de Mbuji-Mayi (ISTM/Mbuji-Mayi); 3 Centre de Recherche Appliquée et Développement (CRAD), Kinshasa, RDC; 4 Centre de Recherche en Epidémiologie, Biostatistiques et Recherche Clinique, Ecole de Santé Publique Université Libre de Bruxelles, Brussels, Belgique; 5 Centre de Recherche en Politiques et Systèmes de Santé-Santé Internationale, Ecole de Santé Publique Université Libre de Bruxelles, Brussels, Belgique

**Keywords:** Planification familiale, méthodes contraceptives modernes, contraceptifs modernes, Family planning, modern contraceptive methods, modern contraceptives

## Abstract

**Introduction:**

l’objectif de cette étude était de déterminer la prévalence contraceptive moderne et les barrières à l’utilisation des méthodes contraceptives modernes chez les couples de la Zone de Santé Dibindi, à Mbuji-Mayi en République Démocratique du Congo.

**Méthodes:**

de Mai à Juin 2015, nous avons réalisé une étude descriptive transversale. Nous avons inclus les femmes âgées de 15 à 49 ans, en union maritale, non enceinte au moment de l’enquête, qui habitaient la Zone de santé Dibindi depuis deux ans et ayant consenti librement de participer à l’étude. La collecte des données a été réalisée par interview libre des femmes. La prévalence contraceptive moderne se référait aux femmes en cours d’utilisation, au moment de l’enquête, des contraceptifs modernes. La comparaison des proportions a été réalisée au seuil de signification de 5%. Le test de Bonferroni a été utilisé pour comparer, deux à deux, les proportions des barrières à l’utilisation des contraceptifs modernes.

**Résultats:**

la prévalence contraceptive moderne à Dibindi, en 2015, était de 18,4%. Elle était faible eu égard aux services de planification familiale disponibles dans cette Zone de santé. Plusieurs femmes refusaient d’utiliser les méthodes contraceptives modernes malgré l’information dont elles disposaient à cause de leur désir de maternité, l’interdiction religieuse, l’opposition du conjoint et la crainte des effets secondaires.

**Conclusion:**

l’information suffisante et centrée sur chaque cliente ou son couple, sur la planification familiale, devrait être renforcée de façon à éliminer les fausses croyances, ce qui accroitrait l’utilisation des méthodes contraceptives modernes.

## Abstract (en)

**Introduction:**

This study aimed to determine modern contraceptive prevalence and the barriers to using modern contraceptive methods among the couples in Dibindi health zone, Mbuji-Mayi, in the Democratic Republic of the Congo.

**Methods:**

We conducted a cross-sectional descriptive study from May to June 2015. Nonpregnant married women aged 15-49 years old at the time of the investigation, living in Dibindi health zone for two years and having freely consented to participate in the study were included. Data were collected by open-ended interview of these women. Modern contraceptive prevalence was referred to women who were currently using, at the time of the investigation, modern contraceptives. The comparison between proportions was performed at the significance threshold of 5%. Bonferroni’s test was used to compare, two by two, the proportions of barriers to using modern contraceptive methods.

**Results:**

Modern contraceptive prevalence in Dibindi was 18.4% in 2015. It was low with regard to family planning services available in this health zone. Several women refused to use modern contraceptive methods despite available information because of their desire for motherhood, religious prohibition, opposition on the part of their husband and fear of side effects.

**Conclusion:**

Sufficient client-centered or couple-centered information and family planning information should be strengthened in order to eliminate the false beliefs and to increase the use of modern contraceptive methods.

## Introduction

La planification familiale (PF) est l’une des stratégies de santé dont l’importance est connue dans la réduction de la morbidité et mortalité maternelle [1]. Les grossesses trop précoces, trop tardives, trop nombreuses et trop rapprochées sont responsables de la majorité de complications obstétricales directes, causes de plus de 70% des décès maternels dans les pays à faible revenu [2]. Il existe une corrélation inverse entre mortalité maternelle et prévalence contraceptive (PC): les pays qui ont une PC élevée ont des taux de mortalité maternelle (TMM) plus faibles [2]. Selon Bhutta [1, 3], à elle seule, la PF peut permettre d’éviter 35% de décès maternels dans les pays pauvres, si au moins 60% des femmes en âge de procréer utilisaient une méthode contraceptive (MC). Il existe également une corrélation entre pauvreté et PC; la majorité de pays avec faible PC sont pauvres probablement à cause des charges importantes à assurer dans les familles avec taille de ménage élevée. Cette corrélation suggère que les mesures visant à renforcer l’utilisation des MC dans un pays, contribuent également à améliorer le statut socio-économique des ménages [4]. Hormis cet avantage sur la santé, la PF permet à la femme de s’occuper professionnellement, en vue de contribuer au développement économique de sa famille. Pour l’homme, elle garantit la bonne santé mentale en lui évitant le stress dû à la survenue d’une grossesse non désirée, diminue les dépenses occasionnées par les maladies et lui permet de répondre aux besoins fondamentaux de la famille grâce aux économies réalisées [2]. Dans les pays à revenu élevé, plus de 70% de femmes ont accès à une MC [5]. En Afrique, où la charge de la mortalité maternelle est la plus importante, à peine 24% des femmes en âge de procréer ont accès à une MC moderne [6]. Cette prévalence est encore très faible en Afrique subsaharienne, où elle est de 2,5% en milieux ruraux contre 9% en milieux urbains [6]. En RDC, elle est de 5,4%. La proportion des femmes en âge de procréer, qui y ont un besoin non satisfait en PF, est élevée. Elle était de 24% en 2010-13 [7]. La contraception moderne n’est pas largement utilisée en Afrique Subsaharienne parce que les contraceptifs n’y sont pas disponibles et aussi, culturellement, ils ne sont pas acceptés [2]. En vue d’y améliorer cette utilisation, les agences internationales appuient les structures de santé avec une gamme de contraceptifs. En RDC, l’Association de santé Familiale (ASF), la Santé en milieu Rural (SANRU) et le Fonds des Nations Unies pour la Population (UNFPA) sont parmi les organisations qui appuient ces activités [8]. La Zone de santé (ZS) de Dibindi, à Mbuji-Mayi, dans la province du Kasaï Oriental, est l’une des ZS appuyées en PF, depuis plus de 15 ans [9]. Selon les données du Bureau Central de cette ZS (BCZS), en 2014, toutes les structures de santé offraient ces services, mais jusque fin 2015, la PC restait inconnue. La PC administrative obtenue à partir des données des registres de PF et rapportées à la population en âge de procréer dans la ZS est la seule connue dans la ZS. Pourtant, elle est biaisée; car elle ne tient pas compte des données concernant les femmes qui utilisent les contraceptifs (pilule et préservatifs) en vente libre dans les maisons de vente des médicaments [10]. Par ailleurs, même en tenant compte de cette PC administrative, les contraceptifs ne sont pas utilisés par toutes les femmes en âge de procréer qui en ont besoin. Les raisons de ce non utilisation ne sont pas aussi connues à Dibindi. Déterminer la PC dans cette ZS la plus appuyée de Mbuji-Mayi, permet d’évaluer l’impact de cet appui. Par ailleurs, identifier les barrières à l’utilisation des MC permettra de mettre en place des mesures pouvant aider à y accroitre l’accès. Cette étude a été réalisée en vue de déterminer la PC et identifier les barrières à l’utilisation de MC chez les couples de la ZS de Dibindi.

## Méthodes

### Description du milieu d’étude

La ZS de Dibindi est l’une des 10 ZS urbaines de la ville de Mbuji-Mayi. Elle est subdivisée en 13 Aires de santé (AS). Selon les estimations de 2015, sa population est de 270 423 habitants [10]. L’Hôpital Presbytérien de Mbuji-Mayi est la structure de référence de la ZS. Toutes les structures (13 CS et l’hôpital général de référence) de cette ZS ont intégré la PF et, depuis 2000, elles sont appuyées en PF par l’UNFPA (2000-2004), AXXES (2008-2010) et SANRU (2007-2014) [9]. Les services de PF y sont offerts gratuitement. Toutefois, certains contraceptifs sont vendus dans les maisons de vente des médicaments, en dehors du circuit officiel de l’offre de ces services et du Système National d’Information Sanitaires (SNIS).

### Sélection des sujets

Nous avons inclus les femmes âgées de 15 à 49 ans, en union maritale, non enceinte au moment de l’enquête, qui ont habité la ZS de Dibindi depuis au moins deux ans et ayant consenti librement de participer à l’étude. Celles jugées inaptes, ont été exclues. Le nombre total de femmes recrutées a été déterminé en tenant compte de la prévalence contraceptive de 27% chez les femmes mariées -prévalence administrative de Mbuji-Mayi telle que rapportée par les données de routine du SNIS [10] et une précision de 5%. Avec un taux de non réponse de 10%, la taille minimale de l’échantillon a été de 303 femmes. Les ménages dans lesquels ces femmes ont été recrutées étaient sélectionnés par échantillonnage en strates. Chaque AS était considéré comme strate. Le nombre de ménages par strate était proportionnel au poids démographique de chacune. Dans chaque strate, la répartition des ménages par avenue était non-proportionnelle au poids démographique. Sur chaque avenue, la sélection des femmes a été réalisée par échantillonnage aléatoire systématique des ménages ayant une femme en âge de procréer [11].

### Matériel et méthodes

Notre étude était descriptive transversale. De mai à juin 2015, nous avons récolté les données par interview à l’aide d’un questionnaire (en français et également traduit en Tshiluba) préalablement pretesté et validé. Les interviews étaient réalisées par une équipe d’enquêteurs formés, pour la plupart, des personnels de santé. Au cours de l’enquête, les variables recherchées étaient, outre les caractéristiques socio-démographiques des femmes, les informations sur la connaissance et l’utilisation des MC au cours du mois de l’enquête [11]. Le consentement éclairé et écrit des femmes a été obtenu avant l’administration du questionnaire. Les précautions ont été prises pour que toutes les informations concernant l’identité de la femme soient confidentielles. Nous avons obtenu l’autorisation de la division provinciale de santé avant de commencer la collecte des données [12].

### Analyse des données

Nous avons utilisé les statistiques usuelles (proportion, moyenne et écart-type, médiane) pour décrire notre échantillon. Les barrières à l’utilisation des MC ont été comparées selon quelques caractéristiques socio-démographiques des femmes. La variation des obstacles en fonction de ces caractéristiques a été recherchée par le test de Khi-carré au seuil de signification de 5%. L’intervalle de confiance à 5% (IC95%) a été calculé pour estimer la précision de la PC [11]. Toutes les données ont été encodées sur Excel 2007 et analysées grâce au Logiciel Stata version 13.0 (College Station, TX, USA). Concernant la PC moderne, elle se référait aux femmes en cours d’utilisation, au moment de l’enquête, de l’une ou l’autre des MC suivantes: préservatif, pilule, injectables (depo-provera, Noristerat), implant, dispositif intra utérin (DIU), par rapport au total de femmes enquêtées [13].

## Résultats

### Profil des femmes

Au total, 343 femmes ont été interviewées au cours de cette enquête. Leur âge moyen était de 28 ans (ET: 9 ans; minimum= 15; maximum=49). Près de deux tiers d’entre-elles (60,4%; n=207) avaient 20-34 ans; 16,7% (58) avaient entre 15-19 ans tandis que 22,7% (78) avaient entre 35-49 ans. Près de trois quart de femmes (70,8% ; n=243) étaient dans un lien monogamique tandis que 29,2% (100) étaient dans un lien polygamique. Plus de la moitié de femmes (55,4% ; n=190) avaient un niveau d’étude secondaire; 32,1% (110) avaient un niveau primaire tandis que 12,5% (43) avaient un niveau universitaire. Nonante deux pourcent quatre dixième (92,4% ; n=317) étaient dans une confession religieuse chrétienne; seulement 7,6% (26) étaient musulmanes. Quarante-et-un pourcent quatre dixième (41,4%; n=142) s’occupaient de la vente; 32,1% (110) s’occupaient du ménage; 10,8% (37) avaient un métier libéral, 9,3% travaillaient dans une entreprise publique, privée ou à la fonction publique. Toutes les femmes enquêtées avaient déjà porté, au moins une fois, une grossesse. Concernant la situation de la grossesse précédant l’enquête, 78,7% (270) avaient accouché normalement et donné naissance à un nouveau-né vivant, tandis que 21,3% (73) avaient connu une issue compliquée ou défavorable 9,7% (33) avaient accouché par césarienne; 4,9% (17) avait connu un avortement; 4,5% (15) avaient connu un mort-né tandis que 2,2% (8) de femmes ont perdu leurs nouveau-nés dans la semaine qui a suivi l’accouchement. Cette grossesse précédente était souhaitée par la majorité de femmes (82,0%; n=281). Seules 4,2% (14) des femmes enquêtées étaient sans enfants; 76,1% (261) avaient entre 1 à 5 enfants; 18,9% (65) avaient entre 6 à10 enfants tandis que 0,8% (3 femmes) avaient au moins 11 enfants. Le nombre médian d’enfants par femme enquêtée était de 3 (minimum=1 et maximum =12). Le nombre d’enfants désirés était variable; 65,9% (226) de femmes préféraient avoir 6 à 10 enfants; 20,6% (71) désiraient avoir 2 à 5 enfants tandis que 13,5% (46) désiraient avoir plus de 11enfants. Le nombre médian d’enfants désirés par les femmes était de 7, variant entre 2 et 18. L’écart inter génésique (EIG) médian, chez les femmes qui avaient au moins deux enfants (266), était de 24 mois (minimum=12 mois ; maximum=60 mois). Près de trois quarts (78,0% ; n=207) de femmes avaient un EIG entre 18 à 36 mois ; 15,2% (41) avaient un EIG entre 37-60 mois et 6,8% (18) avaient un EIG inférieur à 18 mois.

### Information et connaissance des méthodes contraceptives

**Figure 1:**
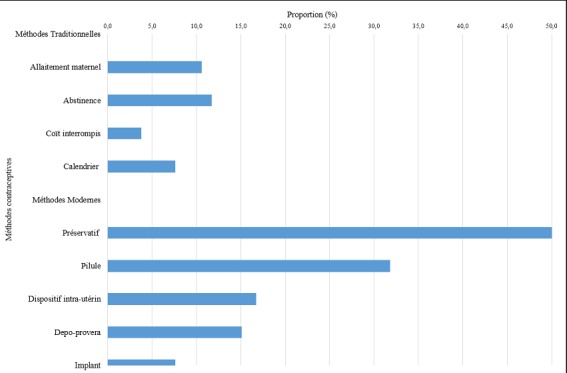
Méthodes contraceptives connues de femmes de la ZS de Dibindi à Mbuji-Mayi, 2015

Dans le Tableau 1, il apparait que trois quarts (77,0%; n=264) de femmes enquêtées avaient déjà entendu parler, au moins une fois, des MC. La majorité de celles informées (63,6%) l’étaient principalement à partir des structures de santé; 20,5% l’étaient à partir des amis; seulement 7,6% des femmes étaient informées à partir de la radio et la télévision. La proportion de femmes informées à travers l’église et les ONG étaient respectivement de 4,5% et 1,5%. Pour les femmes informées (264), les MC avaient l’avantage d’espacer les naissances (67,8%), éviter la grossesse (23,1%), limiter la naissance (2,3%) et 1,5% estimaient que l’utilisation des MC permettait d’éviter les maladies; 1,9% (5) de femmes ignoraient les avantages de l’utilisation des MC (Tableau 1). Dans la Figure 1, il apparait que de toutes les MC modernes, le préservatif (53,0%), la pilule (31,8%) et le dispositif intra-utérin étaient les plus connus. L’abstinence périodique (11,7%), la MAMA (10,6%) et le calendrier étaient les méthodes traditionnelles les plus connues. D’une manière générale, les femmes étaient plus informées des méthodes modernes qu’elles ne l’étaient pour les méthodes traditionnelles (p<0,001).

**Tableau 1:** Informations sur les MC et importance de la PF selon les femmes de la ZS de Dibindi à Mbuji-Mayi, 2015

Paramètres	% (n=343)
**Information sur MC**	77,0
**Sources d’information sur MC†**	
CS/hôpital	63,6
Amis (voisin)	20,5
Radio/télévision	7,6
ONG	1,5
Eglise	4,5
Autres	2,3
**Importance des MC†**	
Espacer les naissances	67,8
Eviter la grossesse	23,1
Limiter les naissances	2,3
Eviter les maladies	1,5
Autre (à préciser)	3,4
Ne sait rien	1,9

† n=264

### Utilisation des méthodes contraceptives

Au total, 99 femmes enquêtées étaient en cours d’utilisation des MC au moment de l’enquête (Tableau 2). Parmi celles-ci, 63 utilisaient les méthodes modernes (63,6%) alors que 36 utilisaient les méthodes traditionnelles (36,4%). Considérées par rapport aux femmes informées des MC, la proportion des femmes utilisant toutes les MC était de 37,5%. Cependant, en considérant toutes les femmes enquêtées, cette proportion était de 28,9%. La prévalence contraceptive moderne chez les femmes en âge de procréer de la ZS de Dibindi était de 18,4% (IC95%: 14,6-22,8%). Dans cette ZS, le préservatif était la MC la plus utilisée par les femmes enquêtées en 2015. Il était suivi de la pilule (7,1%) et le Dépo-provera ou Noristera (6,1%). Les spermicides, le DIU et les implants étaient les méthodes rarement utilisées. Concernant les méthodes traditionnelles, c’est le calendrier qui était utilisé par plus du quart (25,1%) de femmes; l’abstinence périodique (2,0%), la MAMA (3,0%) et le coït interrompu (5,1%) étaient rarement utilisés.

**Tableau 2:** Méthodes contraceptives utilisées par les femmes de la ZS de Dibindi, 2015

Méthodes contraceptives utilisées	% (n=99)
**Modernes**	
Préservatif	39,4
Pilule	7,1
Stérilet/DIU	2,0
Spermicide	1,0
Ligature	2,0
Dépo-provera/Noristera	6,1
Implant	3,0
Ligature tubaire	4,0
Traditionnelles	
Allaitement maternel	2,0
Abstinence périodique	3,0
Coït interrompu	5,1
Calendrier	25,3

### Obstacles à l’utilisation des méthodes contraceptives modernes

**Figure 2:**
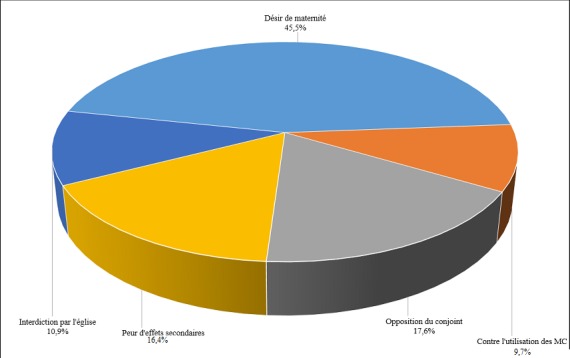
Barrières à l’utilisation des méthodes contraceptives modernes chez les femmes informées de la PF à Dibindi

Dans la Figure 2, il ressort que, chez les femmes informées sur la PF (264), les motifs de non utilisation des MC étaient variables. Près de la moitié de femmes (45,5%) ne les utilisaient pas à cause de leur désir de maternité; 17,6% n’utilisaient pas les MC à cause de l’opposition de leur conjoint; 16,4% avaient peur des effets secondaires des MC (pilule et contraceptifs injectables); 10,9% des femmes n’utilisaient pas les MC parce qu’interdites par leur confession religieuse et 9,7% ne les ont pas utilisées par manque de connaissance de leurs avantages. La Figure 2 présente la répartition des femmes informées de la PF selon les obstacles à l’utilisation des MC modernes. Dans le Tableau 3, la répartition des obstacles à l’utilisation des MC était différente entre les femmes, lorsqu’on tient compte de quelques-unes de leurs caractéristiques gestité et issues de la grossesse précédente mais pas en fonction de leur âge. Chez les femmes de moins de 20 ans, bien que près d’un cinquième (19,2%) ait refusé d’utiliser les MC par peur des effets secondaires, le motif principal de ce refus était le désir de maternité (69,2%). Chez les femmes de 20 à 34 ans, 4 femmes sur 10 avaient refusé les MC à cause de leur désir de maternité; près de 60% l’avaient fait pour d’autres raisons, notamment: l’opposition du conjoint (20,7%), la peur des effets secondaires (14,4%), l’interdiction de l’église et l’avis défavorable de la femme. La proportion des femmes ayant refusé d’utiliser les MC pour raison de désir de maternité était similaire entre les femmes de 20 à 34 ans (41,4%) et celles de 35 ans ou plus (39,3%). Dans cette dernière tranche, outre le désir de maternité, les autres raisons étaient par ordre d’importance: la peur des effets secondaires (21,4%), l’interdiction par l’église et l’opposition du conjoint (14,3%) ainsi que l’avis défavorable de la femme. Bien que cette répartition des obstacles fût variable selon l’âge des femmes, elle n’était pas statistiquement significative. Concernant la gestité, la proportion des femmes ayant refusé d’utiliser les MC pour désir de maternité avait varié selon le nombre de grossesses; elle diminuait alors que le nombre de grossesse augmentait (p=0,032). Chez les primigestes, le désir de maternité était le motif principal (54,7%) du refus des MC. Par contre, chez les femmes qui avaient 2-4 grossesses, plus de la moitié de femmes avaient des raisons autres que le désir de la maternité (41,4%): opposition du conjoint (25,9%), peur des effets secondaires des pilules et contraceptifs injectables (19,0%), avis défavorable vis-à-vis des MC et interdiction par l’église (6,9%). Chez celles ayant eu 5 grossesses ou plus, près de 70% de femmes avaient des motifs autres que le désir de maternité (31,3%): 31,3% avaient refusé les MC par peur des effets secondaires, 15,6% à cause de l’interdiction par l’église, 12,5% à cause de l’opposition du conjoint et 9,6% de femmes n’en voyaient pas les avantages. Les obstacles à l’utilisation des MC variaient également avec l’issue de la grossesse précédente (p=0,003). On notait que les femmes qui ont eu une issue défavorable à la grossesse précédente n’avaient pas utilisé les MC principalement pour cause de désir de maternité (60,0%). Le désir de maternité était deux fois plus important chez ces femmes que chez celles qui avaient accouché normalement à la grossesse précédente. Dans cette dernière catégorie, près de 70% de femmes avaient des motifs de refus d’utilisation des MC, autres que le désir de maternité, notamment: l’opposition du conjoint (26,0%), la peur des effets secondaires (16,4%), l’interdiction par l’église (13,5%) et le manque présumé d’avantages des MC (10,6%).

**Tableau 3:** Barrières à l’utilisation des MC modernes selon quelques caractéristiques démographiques des femmes informées de la PF

Caractéristiques	N (%)	I(%)	II(%)	III(%)	IV(%)	V(%)	p
Age (années)							0,163
<20	26 (100,0)	69,2	3,9	7,7	19,2	0,0	
20-34	111(100,0)	41,4	10,8	20,7	14,4	12,6	
≥35	28(100,0)	39,3	10,7	14,3	21,4	14,3	
Gestité (nombre)							0,032
1	75(100,0)	54,7	12,0	13,3	8,0	12,0	
2-4	58(100,0)	41,4	6,9	25,9	19,0	6,9	
≥5	32(100,0)	31,3	9,4	12,5	31,3	15,6	
Issues de la GP†							0,003‡
Accouchement	130(100,0)	33,7	10,6	26,0	16,4	13,5	
Défavorable	35(100,0)	60,0	5,7	0,0	34,3	0,0	

Désir de maternité=I ; Contre l'utilisation des MC=II ; Opposition du conjoint=III ; Peur d'effets secondaires=IV ; Interdiction par l'église=V ; †=n=127 ; ‡ : Fisher exact ; GP : grossesse précédente ; Issues défavorable= avortement, mort-nés, décès néonatal ou infantile.

## Discussion

Dans ce travail, il a été observé une prévalence contraceptive moderne de 18,4%. Une proportion importante des femmes n’utilisent pas les MC en dépit de l’information dont elles disposent. Pour la plupart, le désir de maternité, l’opposition du conjoint, les interdits de l’église, la peur des effets secondaires et l’ignorance des avantages de la PF restent les aspects biologiques et socioculturels qui limitent l’utilisation des MC. En effet, la PC observée à Dibindi reste au-dessus de la moyenne nationale [7] et Africaine [6]. Elle est près de quatre fois supérieure à celle rapportée au niveau national en RDC et avoisine celle rapportée dans la ville de Kinshasa [7] et dans plusieurs autres villes en Afrique [6]. Comme déjà mentionné plus haut, c’est l’appui des activités de PF, depuis plus d’une décennie, par UNFPA, AXXES et SANRU, qui explique cette prévalence contraceptive plus élevée par rapport à celle qui est observée dans plusieurs autres villes de la RDC. Toutefois, pour autant que toutes les structures de santé représentant les 13 aires de santé de Dibindi aient intégré la PF, cette prévalence reste faible. Cette faible prévalence eu égard à la disponibilité des services de planification familiale est due en plus de la proportion moins élevée des femmes informées de la planification familiale (77,0%) généralement moins importante par rapport à celle rapportée dans plusieurs villes de la RDC et en Afrique [6], à la multitude d’obstacles auxquels sont confrontées les femmes en âge d’utiliser les MC [14–17]. Dans la Figure 2 de ce travail, il a été montré que la majorité de femmes (45,5%), bien qu’informées des MC, ne les ont pas utilisées à cause du désir de maternité [7]. Le nombre médian d’enfants chez les femmes enquêtées était de 3 alors que celui d’enfants désirés était de 7. Cet écart de 4 enfants reste important. Lorsque le désir de maternité a été étudié selon le nombre de grossesses déjà conçues par les femmes, il se dégage que plusieurs raisons, autres que le désir de maternité, justifiaient le non utilisation des MC. Cette information suggère que les femmes commencent à s’intéresser aux MC lorsque le nombre actuel s’approche du nombre idéal d’enfants. Dans ce cas, sans avoir atteint ce nombre et quel que soit leur âge, les femmes utilisent les MC uniquement pour leur avantage lié à l’espacement des naissances plutôt qu’à leur limitation [18–21]. Cette information corrobore les meilleurs écarts intergénésiques (plus de 75% des femmes avec EIG supérieur à 24 mois) observés dans cette population en dépit d’une faible PC moderne. L’opposition de l’homme à l’utilisation des MC par la femme est souvent discutée comme une question de droit relatif à l’autonomie de la femme [22]. Elle est une des explications aux grossesses non désirées, conçues par la femme uniquement sur volonté de son conjoint [23]. Toutefois, au-delà de ce refus du partenaire, il faudra envisager la manière dont les services de santé sont organisés [14–17]. D’abord les considérer: « du couple mère-enfant » exclut la possibilité d’impliquer l’homme dont le rôle est important dans l’utilisation et la qualité de certaines interventions de santé. En effet, dans la plupart de structures de santé, les services sont conçus pour accueillir les femmes et non l’homme. La plupart des activités de PF sont centrées sur la femme, et, il appartient à celle-ci d’informer et convaincre son conjoint pour utiliser les MC [22, 24]. L’opposition des conjoints à laquelle sont confrontées les femmes est probablement synonyme de l’incapacité de certaines femmes à convaincre leurs partenaires à cause des informations insuffisamment reçues dans les services de santé ou trop centrées sur la femme; cette dernière devant jouer l’intermédiaire du personnel de santé dans le ménage [25]. A Lubumbashi, le non implication des hommes dans les activités de PF était associé à un risque plus élevé de non utilisation des contraceptifs modernes par les femmes [25]. Refuser d’utiliser les MC à cause du désir de maternité, interdiction par l’église, opposition du conjoint ou crainte des effets secondaires, ramène en général à la question de la suffisance de l’information sur la planification familiale [2]. Il existe encore beaucoup de fausses croyances autour des MC dans la communauté [7]. Si l’information donnée est superficielle et lorsque celle-ci ne tient pas compte de tous les motifs du refus, les femmes risquent de ne pas les utiliser à cause des fausses croyances. Cette observation suggère qu’au-delà des messages de sensibilisation souvent offert en groupe, des entretiens individuels centrés sur les problèmes de chaque femme ou couple, devraient être organisés de façon, d’une part, à aider les femmes à mieux justifier cette intervention de santé, d’autre part à avoir le choix sur la gamme des MC, ce qui leur permettrait d’utiliser celles qui sont les plus compatibles avec leur situation [2, 9]. Cette étude transversale, réalisée par interview, pourrait avoir certaines limites. Premièrement, il est possible qu’à cause du biais de mémoire, les femmes aient confondu l’utilisation actuelle et passée des MC. Toutefois, le fait d’avoir focalisé l’utilisation des MC au cours du mois de l’enquête, a limité l’impact de ce biais. Deuxièmement, il est aussi possible que le fait d’avoir tenu compte uniquement des obstacles principaux, ait réduit l’ampleur de certains obstacles.

## Conclusion

La prévalence contraceptive moderne à Dibindi en 2015 était de 18,4%. Elle reste faible eu égard aux services de santé de planification familiale disponibles dans la zone de santé. Plusieurs femmes refusent encore d’utiliser les MC malgré l’information qu’elles ont sur planification familiale. Le désir de maternité, l’interdiction par l’église, l’opposition du conjoint et la crainte des effets secondaires des MC étaient les obstacles à l’utilisation des MC les plus évoqués. L’information suffisante et centrée sur chaque cliente ou son couple, sur la planification familiale, devrait être renforcée de façon à éliminer les fausses croyances, ce qui accroîtrait l’utilisation des MC.

### Etat des connaissances actuelle sur le sujet

- La planification est une intervention à haut impact pour la réduction de la mortalité maternelle et infantile;
- La prévalence contraceptive reste très faible dans les pays à faible revenu;
- Les services de planification familiale ne sont pas toujours disponibles là où les femmes en ont besoin.

### Contribution de notre étude à la connaissance

- La prévalence contraceptive moderne reste faible à Dibindi en dépit de la disponibilité des services de planification familiale;
- Les femmes utilisent moins les contraceptifs modernes tant qu’elles n’auront pas encore atteint le nombre idéal des enfants;
- Si les hommes ne sont pas impliqués dans les services de planification familiale, ils constituent, pour les femmes, une barrière à l’utilisation des contraceptifs modernes.
